# On dementia, duties, and daughters. An ethical analysis of healthcare professionals being confronted with conflicts regarding filial duties in informal dementia care

**DOI:** 10.3389/fpsyt.2024.1421582

**Published:** 2024-10-01

**Authors:** Vildan Dogan, Marija Taneska, Gabriela Novotni, Svetlana Iloski, Antoni Novotni, Vesna Dimitrova, Miloš Milutinović, Ljubisha Novotni, Anne Weber, Boban Joksimoski, Ivan Chorbev, Shpresa Hasani, Andrea Ivanovska, Timo Grimmer, Julia Fischer

**Affiliations:** ^1^ Clinic for Psychiatry and Psychotherapy, Center for Cognitive Disorders, TUM School of Medicine and Health, Technical University of Munich, Munich, Germany; ^2^ Institute for Alzheimer’s Disease and Neuroscience, Skopje, North Macedonia; ^3^ Faculty of Medicine, University Cyril and Methodius, Skopje, North Macedonia; ^4^ Faculty of Computer Science and Engineering, University Cyril and Methodius, Skopje, North Macedonia; ^5^ Hannover Institute for Philosophical Research, Hannover, Germany

**Keywords:** dementia, informal caregiving, filial duties, ethical conflict, healthcare professionals, North Macedonia, caring democracy

## Abstract

**Background:**

Existing literature on moral conflicts that healthcare professionals encounter in dementia care has explored, amongst others, issues related to autonomy, decision-making capacity, privacy, and more. Notably, conflicts related to healthcare professionals who support informal dementia caregiving and who are confronted with family members being overburdened with their care responsibly remains an underexplored topic in the current literature, particularly in the context of Low-and Middle-Income Countries. The present paper introduces such an encounter, presenting an ethical case analysis of a conflict that occurred during a larger research project conducted in North Macedonia.

**Case to be studied:**

Due to the absence of formal care services that could have relieved an overburdened family caregiver, healthcare professionals felt compelled to reach out to the uninvolved adult daughters, requesting them to participate in their parents’ care. Wondering about whether their reaching out to the daughters might count as an attempt of pressure and undue interference, professionals conflicted over the appropriateness of their action. This paper follows up on their concern, ethically assessing the professionals’ action. To answer the question on whether the healthcare professionals acted appropriately or not, and to what extent, theories of filial duties are applied, embedding their action in the larger context of dementia care in North Macedonia.

**Results and conclusion:**

It is argued that the lack of formal care services in North Macedonia is of utmost relevance to the conflict. Thus, the conclusion is that the ethical inappropriateness of the case is to be located not so much with the action of the healthcare professionals but with the state because of its failure to provide professional care services that allow healthcare professionals to take ethically sound actions to counteract overarching burdens that family members face when providing informal dementia care

## 1 Introduction

In parallel to the ongoing scholarly and political discourse on how care responsibilities are to be distributed between the family and the state (1–5), in most countries, dementia care is provided mainly by female family members (6–9). While performing informal dementia care may be experienced positively, it frequently comes with negative effects for family caregivers (10–13). They often experience significant physical, emotional, and financial burden due to providing informal care (6, 14–16). Also, they often face social isolation as the demands of caregiving limit their ability to engage in social activities (17, 18). Lack of sufficient formal support services is known to exacerbate stress, depression, and other negative effects experienced by informal family caregivers (13, 19–21).

In North Macedonia, dementia care is also mainly provided by female family members, such as daughters (8). Located in the central Balkans region, it is a middle-income country with a population of nearly two million. Ethnic Macedonians make up 60% of the population, followed by ethnic Albanians (21%) (22). Around 17% of the population is over 65 years of age (22). Currently, about 28,279 people live with dementia, with an expected increase of 166% by 2050 (23). In North Macedonia, stigma and negative attitudes towards dementia prevail. Dementia is still referred to as ‘sclerosis’, a term often used as an offense. The stigma arises from perceiving persons living with dementia as deviations from the norm (24). Dementia is not recognized as national policy priority and the formal dementia care system in North Macedonia faces significant deficits in professional care provision (25, 26). The latest statistical review of social care users in 2020 stated that only about 1,500 adults (of all ages and conditions) are in institutional care (27). Most persons living with dementia are cared for by their family members at home. As the availability of formal care services is limited, family members, often adult daughters (8), are forced to provide dementia care without professional support (25, 27, 28). This negatively affects their quality of life (25, 28). Insufficient provision of formal care services has been pinpointed as the most significant predictor for reduced quality of life among informal dementia caregivers in North Macedonia, closely followed by elevated burdens and depression levels (28).

The NOMAD (*North Macedonia Interprofessional Dementia Care*) project was developed against the backdrop of inadequate dementia care structures in North Macedonia. The overall objective was to implement an interprofessional model of dementia care and to evaluate the potency of the model to improve the living conditions for families affected by dementia (8). The dementia care model employed interprofessional teams each comprised of one social worker and one nurse, referred to as ‘mobile memory teams’ (MTs). The MTs conducted home visits to individuals living with dementia and their family caregivers, residing in Skopje and surrounding rural areas. During the home visits, the MTs assessed their living conditions and identified their needs in domains such as physical health, mobility, environment, and psychosocial well-being. MTs were associated with general practitioners with whom they developed and implemented a comprehensive care plan that focused on non-pharmacological measures. Care plans and measures aimed at improving the living situation of both the individual living with dementia and their family caregiver(s). Packages included, amongst others, home safety suggestions to mitigate potential hazards and risks in the home environment, guidance on self-care practices for informal caregivers, anti-stigma education, assistance for financial aid and care allowances applications, guidance for managing challenging behaviors and communication difficulties, as well as support for adapting to changes in the individual’s abilities. The model’s effectiveness was evaluated through a cluster-randomized control study involving a total of 120 families (60 per trial arm). Various questionnaires were administered to collect differences in outcomes between both groups – to the end of evaluating the effectiveness of the intervention. The detailed study results were published. They showed the effectiveness of the intervention in improving the living condition of both the person living with dementia and their family caregivers (8).

During the NOMAD project, one MT encountered a conflict. The conflict arose as a result of missing formal care provision equipped to unburden an overburdened family caregiver. It was an older husband caring for his wife diagnosed with dementia. The informal caregiver was frail himself and experienced poor health. Their living conditions were poor, and he struggled to cope with his wife’s advancing dementia. Due to the absence of professional care services that could have relieved the overburdened informal caregiver, MT members contacted the adult daughters, who had not been involved in their parents’ care. The MT aimed at getting the daughters to step in to help. Wondering about whether their reaching out to the daughters might count as an attempt of pressure and undue interference, one MT member conflicted over the appropriateness of their action. Eventually they decided to proceed.

This paper follows up on the concern of the MT member. To address the question on *whether the MT acted appropriately or not, and to what extent*, MT’s action is discussed in light of ethical theories on filial duties. Filial duties refer to obligations and responsibilities that adult children are expected to have towards their parents according to certain moral principles rooted in cultural and societal norms (29). In everyday moral concepts filial duties, as in the present case the claimed duty to care for one’s older parents, are most often accepted uncritically. A sense of reciprocity for the care and support provided by parents throughout one’s life is suggested (2, 30, 31). This also applies to North Macedonia. Here, caring for parents in advanced age is considered the natural, expected, and moral order of things (32). Not fulfilling this filial duty can result in feelings of guilt, shame, and public humiliation, referred in Balkan countries as ‘loss of face’ – the erosion of one’s public image and status (32). The impact of these societal expectations is substantial. Surveys show that almost 80% of respondents in the Balkans felt pressured to provide informal care, even at the expense of their careers (33). Given gendered expectations, especially daughters are perceived as ‘natural caregivers’ (33, 34).

The MT’s conflict is operationalized as a moral conflict in which the MT had to choose between either leaving the father as an overburdened caregiver on his own or imposing the burden of informal caregiving on the daughters who were not involved in the mother’s dementia care until then. Moral conflicts may be conceptualized as situations “involving a clash of moral values within the practitioner, among practitioners, and/or between practitioners and patients, concerning what was the morally right action to take” (35). The literature shows that such conflicts are commonly encountered by professionals across the whole dementia trajectory. They are influenced by the condition’s complexities as well as by cultural and religious beliefs (36, 37). Often such conflicts are accompanied respectively caused by scarcity of resources, such as staff and time, and could be avoided under more resourced circumstances (36, 38). Scholars have studied moral conflicts using the concept of moral distress. This may be defined as a phenomenon that combines “[1] the experience of a moral event, [2] the experience of ‘psychological distress’, and [3] a direct causal relation between [1] and [2]” (39). The recent literature debates about what it means to experience moral distress (40–42). Many authors stress that the frequency and severity of moral distress are high and a serious problem. They are working on effective interventions to mitigate moral distress (43, 44). Others, however, also point to positive effects of moral distress. They argue that it can draw the attention of professionals to systemic issues and deficiencies within the healthcare system and motivate them to advocate for better care standards and policies. This advocacy can lead to improvements in care provision that benefit both healthcare professionals and those in need of care and support (45–47).

The ethical case analysis aims to contribute to the literature that deals with moral conflicts in dementia care experienced by healthcare professionals. To the best of current knowledge, conflicts that healthcare professionals who support informal dementia caregiving (as carried out by the NOMAD MTs) encounter when being confronted with overburdened family members remain an underexplored topic in the current literature. Engaging with the ethics of filial duties, the assessment of the MT’s action hinges on whether filial duties warrant requesting adult daughters to care for their parents or whether it is ethically problematic to call upon adult children to provide unpaid informal care for their parents. By integrating the ethics of filial duties with the literature on moral conflicts and moral distress in dementia care, a connection is established to the debate on caregiving responsibilities and informal caregiving burden. The present analysis will hold systemic deficiencies accountable for moral conflicts, illustrating the inherent political nature of this ethical quandary and many others. In what follows, an overview of how the conflict that this paper departs from came to attention is provided, before delving into the conflict’s ethical quandary.

## 2 Identifying the conflict at hand

To gain deeper insights into the implementation of the care model introduced by the NOMAD project, semi-structured interviews were conducted with all MT members after the delivery of the intervention was completed. The interviews with the MTs were undertaken with the purpose of gathering insights crucial to the real-world application of the care model, fostering its integration within healthcare practices. All six team members (three social workers and three nurses), five of which were females, were interviewed. Their average age was 40 years old and they had an average working experience of 18 years. Individual interviews were conducted online in autumn 2023, with each session lasting approximately one hour. MT members were invited to share their experiences with the intervention and to review, from their professional perspectives, the care model that they pioneered implementing. They were asked to describe the care packages they developed in collaboration with the GPs, to elaborate on the measures they implemented or recommended to family caregivers, and to report on their collaboration with the GPs. Furthermore, MT members were invited to share their perspectives on what they consider to be the greatest challenges in dementia care in North Macedonia, and how the care model may contribute to address these problems.

The interviews were conducted in Macedonian, the native language of the MTs, by one of the first authors of this paper, Taneska, M., who, too, is a native speaker. Considering recommendations on cross-language qualitative research (48), the interviews were transcribed in original language and afterwards translated into English. This was done to make the interview data accessible to the research team members from Germany who do not have a command of the Macedonian language. Taneska, M., who is also proficient in English, translated the transcripts.

All MT members consented to being interviewed. The whole NOMAD study was approved by the Ethical Committee of the Medical Faculty at the University Ss Cyril and Methodius in Skopje, North Macedonia (Ref Number:03-1260/5). The study was conducted in compliance with European data protection guidelines.

When familiarizing with the data, a situation described by members of one MT caught attention. It is crucial to clarify that the original research objectives did not encompass an examination of ethical concerns. However, as this unanticipated issue surfaced, it seemed worthy of closer analysis. In the following, the situation is described, using quotes from the interviews to strengthen the transparency of the work. The case was not identified by applying qualitative research methodology on data analysis. The case to be presented stood out because of the difference in between the accounts of the healthcare professionals involved in the situation.

One MT was assigned to a married older couple. The wife, who was living with dementia, was cared for by her husband. Together, they were living in a rural area near Skopje, in a family house that was in substandard condition. The wife was severely impacted by cognitive decline, which had manifested over the past few years. Her cognitive abilities had deteriorated to the point where she experienced significant memory loss. Her speech was slurred and barely intelligible. Physically, she was frail, emaciated, and required assistance with activities of daily living, including personal hygiene. The wife also exhibited wandering behavior, posing safety risks. Her condition had rendered her incapable of engaging in activities she previously enjoyed, such as reading, writing, or using a phone. Her progressive decline resulted in her becoming almost entirely dependent on her husband.

The husband was primarily responsible for the care of his wife. He was struggling significantly with this role. MT members described him as worn out, confused, anxious, and overwhelmed by the situation. His health was also impaired, adding to the difficulty of providing care. He was worried about the family’s situation and seemed to be under immense stress.

Given that the care needs of the wife were not met by the husband who was found to be heavily overburdened with his caring responsibility, the MT regarded support for the couple to be urgently needed. Faced with a lack of formal dementia care options, the MT members reached out to the couple’s three adult daughters, who had previously not been involved in the care of their mother. Contact was made by telephone. During the telephone conversation with one of the daughters, the MT emphasized the need for additional help in caring for both parents. In subsequent visits, one of the daughters was always present and actively participated in the program, providing much-needed support to both the patient and her husband.

*Well, we had one specific case where the patient lived with her husband. They had three [adult] daughters, and the living conditions weren’t really … it was untidy, the daughters rarely visited. Apart from the problems of the wife [meaning the person living with dementia], the health of her husband who was her caregiver wasn’t good. So, simply, he also met the criteria of someone who needed help. So, there we intervened, and we called the daughters and there was a positive outcome, so with each consecutive visit, although we weren’t precise, the home was tidy. The patient looked neat, so…* (Translated quote from the nurse who was a member of the MT)

As explained in the introduction, the MTs were given authorization to implement non-pharmacological measures as part of the NOMAD intervention, which, in their professional opinion, were equipped to help improve the living situation of the families. In the specific case, the MT members gained consent from the family caregiver (meaning the husband of the woman living with dementia and the father to the daughters) to contact his adult children. They did not explicitly obtain consent from the daughters to be contacted, nor were the daughters listed as emergency contacts.

While accounting for the case, one MT member started wondering about the appropriateness of their interference with the daughters.

*I don’t know if they [meaning the adult daughters] experienced it as a threat … maybe they thought … but anyway, someone was going into the home and asking about their condition, and the daughters were more involved.* (Translated quote from the nurse who was a member of the MT 1)

While both MT members felt relieve because of witnessing that the living situation of the couple had improved due to the involvement of the adult daughters, only one MT member also felt discomfort because of reaching out the daughters to impose the burden of informal caregiving on the daughters who were not involved in the mother’s dementia care until then. Her team partner showed no discomfort during the interviews.

*From the daughters who were somehow aside because they were really busy, afterwards they were really involved in all of that, in their mother’s care. And we explained to them that their dad is unwell and what would happen if God forbid and that it’s better for them to be there than not call him at all and his health to get worse and etc.* (Translated quote from the social worker who was a member of the MT 1, talking about the same case)

That one MT member problematized their interference with the adult daughters and the other did not shows that a situation can be perceived as both a conflict and no conflict at the same time – which calls for a closer examination of the case as to whether the MT did or did not act ethically appropriate by reaching out to the uninvolved daughters to get them involved in the care of their parents.

## 3 Discussing the conflict at hand

The crucial question to be addressed is about the appropriateness of the MT’s reaching out to uninvolved adult daughters and requesting them to participate in their mother’s care. It is to be acknowledged that the outcome of the MT’s intervention, as observed by the MT, was effective in that sense that the mother received better care and the overburdened father as caregiver received support and relief. Authors have argued that actions may be considered morally right that prove efficacious in practice (49). From this point of view, the MT acted morally right by involving the uninvolved adult daughters. However, this line of arguing dismisses the interests of the daughters too quickly and fails to problematize the absence of formal care provision that caused the conflict in the first place.

The case described reveals a triadic conflict of interests and rights as also observed in other care constellations (50). Referring to their job assignment and work ethos, the MT members have an inherent interest in the best possible care for the individual living with dementia, in this case the woman living with advanced dementia. Complementarily, the woman living with dementia herself has a fundamental right to receive a form of care that at least prevents further harm and ideally supports well-being according to her condition. The third party, here the husband as well as the daughters, supposedly have an interest in the best possible care for the wife/mother due to their relationship, but also have the right to physical integrity and self-determination including (partial or full) detachment from the care responsibilities. Especially when personal dignity is compromised, such conflicts can hardly be resolved by individually balancing the conflicting interests and rights. Due to the complexity of care settings, it is neither possible to justify asymmetric duties, including filial obligations, nor can their validity be claimed beyond the individual case. The moral conflict surfaced in the study fundamentally highlights the precarity of care situations in private households. It also illustrates that both, the well-being of those in need of care and the well-being of informal caregivers, are exposed to considerable (health) risks. Against this backdrop, it can be argued that facing such fundamental ethical and practical quandary, the state is constitutionally obliged to take responsibility in form of providing professional care services and considering the needs of all involved parties (51, 52). There are states that are fulfilling their responsibility, providing professional care services such as respite care, memory clinics, day care centers, telemedicine services, companionship services for both the individual living with dementia and their family caregivers, and/or mobile care services that offer assistance with activities of daily living, such as helping with bathing, dressing, grooming, eating, and toileting (53–64). In most states, however, professional care services are not or only insufficiently available (65), such as North Macedonia.

The lack of formal care services in North Macedonia is of utmost relevance to the conflict faced by the MT. As described in the introduction, dementia care in North Macedonia is characterized by significant deficits (23, 26–28). The failure to provide appropriate formal dementia care services is directly related to the exploitation of family members as unpaid caregivers – a phenomenon common not only in low- and middle-income countries, such as North Macedonia, but also in high-income countries, such as Germany (66). The lack of formal care services and the exploitation of family caregivers is linked to political debates that are premised on functional understandings of family members as caregivers whose right to partial or full detachment from care responsibilities carries little weight (67, 68). Such debates often apply conceptions of filial duties to discuss “how responsibility for the care of the aged should be divided between the family and the state” (2). Conceptualizations of filial duties, as explained in the introduction, depart from suggesting obligations and responsibilities of adult children towards their parents, including the duty to care for one’s parents (29). Filial duties, hence, are often used as a counterargument to the state’s responsibility to provide care, framing care as a family matter (69). Applying this argument to the present case, one could claim that the conflict is unrelated to the failure of the North Macedonian state to provide professional care structures as the daughters would have been obliged to care for their parents anyway. In the following, this claim is challenged. By drawing on different accounts of theories that try to substantiate filial duties, a basis is provided to ethically assess whether the MT acted appropriately or not, and to what extent. Contrary to what might be expected, this analysis will show that by closer examination theories on filial duties do not contribute to releasing the state of its responsibility to provide care but does the opposite.

### 3.1 Applying theories on filial duties to the present case

The so-called *debt theory* is, to the current knowledge, the oldest attempt that tries to establish and substantiate filial duties, dating back to Aristotle. According to debt theory, adult children are viewed as debtors of their parents and are morally obliged to settle the debts incurred through their upbringing (29, 70). This includes intensive care, financial sacrifices, adjustments to career plans, and giving up time-consuming hobbies (71). However, this theory faces widespread criticism, mainly for two reasons (2, 72, 73). Firstly, the theory fails due to the misconception of a contract between parents and children. A debt relationship must be preceded by a type of contract in which the future debtor (in this case children) voluntarily agrees to become a debtor. If applied, this would mean that children would get into debt by being born and by being cared for as minors. However, unborn children do not voluntarily agree to a contract to be born and minor children do neither voluntarily agree to a contract to be cared for (72, 73). Secondly, the theory is not equipped to solve the problem of the non-existent possibility to quantify filial debts. The concept of debt involves a clear obligation to repay a specific fixed amount of certain goods. Without a fixed amount, a debt can never be settled. Most goods that adult children are said to owe their parents are, however, defined by an unquantifiable nature (72, 73). Emotional support – as one example for a good that parents (are expected to) provide for their children throughout childhood – cannot be easily measured and repaid. Ultimately, dept theory turns out to be of no help for this analysis as the theory has been largely disapproved by the scholarly literature in that the concept of debt cannot be used to substantiate filial duties.

A second theoretical lens through which filial duties are often considered is the so-called *gratitude theory*. Gratitude theory posits that children are obligated to be grateful to their parents because of past parental achievements. This perspective suggests that filial duties arise from a sense of gratitude for past services (74–77). This theory, however, is being criticized, too. Numerous authors argue against understanding filial duties solely as duties of gratitude (2, 31, 78). They contend that not everyone feels comfortable receiving reciprocal acts, as intrinsic motivation drives such actions, done to benefit someone rather than to receive something in return. Consequently, the initial benevolence becomes doubtful, and expressing the depth of gratitude becomes challenging, as children may always feel inadequate towards their parents (2). Furthermore, the extent of duties of gratitude depends on the discomfort, exertion, and sacrifice involved in raising the child, factors that are challenging to measure (2). What is more, the theory faces criticism for its complexity, as basic duties are considered universal, but authors argue that obligations only apply if parents were proficient in parenting in the past (78, 79). According to a widely shared understanding, it is the parental duty to provide their children with appropriate goods (31). Conversely, if there is nothing for the offspring to be grateful for due to insufficient care in the past, the theory can’t be applied accordingly. This raises the question of how being raised well is defined and by whom. It remains unclear why children would owe parents gratitude for the fulfillment of parental duties. Consequently, only children whose parents provided extraordinary services would be obliged to have duties emerging from gratitude. The lack of clarity on what constitutes extraordinary services and who determines the circumstances contributes to the problematic nature of this theory (31). Moreover, parental care does not constitute an advance performance, as a child has not demanded this performance. Conversely, what a child has given to its parents does not generate any obligation for gratitude on their part either (78). Although parents and children can be grateful to each other, gratitude is not the subject of a duty but rather a piety (79, 80). In sum, it appears that gratitude theory, if at all, only succeeds in establishing filial duties for those cases in which parents provide extraordinary parental care as defined by their children.

A third theory - known as *the friendship theory -* bases filial duties on love and affection between parents and adult children (72). In contrast to gratitude theory that addresses the relationship between children and their parents during childhood, this model seeks to substantiate filial duties with regard to the present relationship between parents and their adult children (2). Dixon (1995) explains that the relationship between parents and adult children gives rise to the duties among friends and is analogous to friendships in terms of moral dimensions (81). Critics of the theory problematize that characterizing parent-child relationships as friendships seems forced (31). They criticize that the parent-child relationship is unique and not comparable to conventional friendships. Also, it is not possible to choose one’s parents. Children are stuck with their obligations towards their parents in a way that can’t be transferable to duties of friendships, as those are based on different factors (e.g., simultaneous interests) (2). Proponents of the theory, however, stress the theory’s advantages over debt and gratitude theory. For instance, the friendship model of filial duties can explain why duties do not differ depending on parental sacrifice and why reciprocal performance can never be discharged (2).

So far, it appears that substantiating filial duties requires an understanding of the unique parent-child relationship, making it a complex and context-dependent issue. It turns out that the question of duties depends centrally on the perspective of the adult children, on whether they acknowledge their parents’ parental care as extraordinary (resulting in filial duties due to reciprocity) or on whether they view their parents as friends (resulting in filial duties due to the present relationship rather than to the past relationship during childhood). This means that through the lens of gratitude theory as well as through the lens of friendship theory, the answer to the question on whether the MT (by reaching out to the daughters) acted appropriately or not depends on the perspective of the daughters. The present data does not allow us to sufficiently explain why the daughters initially did not participate in their parents’ care and then did so at the request of the MT. No legit conclusions can be drawn about their agency, that is about their power to turn down the MT’s request. However, the introduction, outlines the societal expectations regarding the moral obligation to care for one’s family members that prevail in North Macedonia, putting the daughters under pressure to comply to such expectations. What is more, dementia stigma remains a major issue in North Macedonia, as also pointed out in the introduction. Embarrassment, thus, could be another reason contributing to why the daughters got involved. Taking these contextual factors into account, it is to be emphasized that the taking up of care responsibilities by the daughters must not necessarily be interpreted as an acknowledgement in that sense that they agree to have filial duties towards their parents.

A fourth theory is known as *special goods theory* (2). Special goods theory emphasizes the uniqueness of family relationships, introducing the concept of special goods that only parents and children can exchange. This school suggests that the value lies in the affirmation of the relationship through unique goods, which is why those can’t be delegated to third parties (e.g., nurses). The so-called generic goods can be provided by anyone and not merely the parents or children. Special goods, on the other hand, are explained to be more profound, as parents often share common traits with their offspring and can identify with them, leading to a special understanding. The sense of continuity due to getting along with the whole life development is of value (2). For example, the care for the parent can be outsourced, but the joy of a visit from the children cannot. Special goods theory thus suggests that by reaching out to the daughters the MT did not act appropriately as the MT did not ask the daughters to provide a special good but to get involved in hands-on dementia care – which is not considered to be a filial duty according to the theory. This assessment is, however, of little insight given the theory’s shortcoming. The theory grossly ignores the context within which families live. The conflict unfolded in a situation with no instance existent to which the task of caregiving could have been delegated to other than to the daughters. The MT had to choose between either leaving the father as an overburdened caregiver on his own or imposing the burden of informal caregiving on the daughters who were not involved in the mother’s dementia care until then. Operating in a vacuum that overlooks the systemic embeddedness of care responsibilities, the theory is not equipped to contribute to answering the question on whether the MT acted appropriately or not as the theory does not allow to take into account that no alternative formal care structures were in place that the MT could have delegated the task of caregiving to.

Taken together, theories on filial duties are not equipped to establish that adult children may be demanded to care for their parents. Filial duties can only be established on a case-by-case basis; they do not justify the state’s failure to provide adequate formal care provision that allow adult children to partially or fully detach themselves from care responsibilities.

That adult children are not to be exploited as unpaid informal care workers to compensate for inadequate professional care structures counts as an insight that needs to be spread among healthcare professionals. As described, one MT member problematized their interference with the adult daughters, whereas the other member took no offense and did not show any sign indicating that she experienced the situation as morally distressing, illustrating that the same situation can be perceived differently by different individuals. This highlights individual differences in perceiving and reacting to moral conflicts and leads to the crucial insight that the absence of moral distress in healthcare professionals does not equate to ethical soundness; neither does its absence mean that the healthcare environment is free from systemic deficiencies. It is important that healthcare professionals become more conscious towards the problematic nature of exploiting family caregivers as only then they might experience relevant situations as moral distress – which, as explained in the introduction, might motivate them to become politically active, advocating improvements in systemic dementia care provision (45–47).

Being thrown into the conflict of having to choose between either leaving the father as an overburdened caregiver on his own or imposing the burden of informal caregiving on the daughters who were not involved in the mother’s dementia care until then, the MT followed a pragmatic approach that was necessary in view of the mother’s lack of care and the excessive demands on the father as caregiver. Such approaches are common in North Macedonia and other Balkan countries. Here, healthcare professionals are used to seek alternative solutions to accomplish tasks, making informal institutions, networks, and practices common for service provision in the Balkan region. Such informality is about the attempt to ‘getting a job done’, about recognizing the necessity of utilizing informal practices amidst the shortcomings of public institutions, particularly in health and social care (82). However, because of its effectiveness, informality contributes to systemic deficiencies being concealed, as it supposedly looks as if no systemic change is needed at all as solutions are being found. In the particular instance of the present case, the action of the MT was morally sound, but only because they had no other choice. This reveals a dual nature to their action: while morally sound, it remains ethically problematic as it is not generally appropriate to involve uninvolved adult children in their parents’ care. Consequently, the moral conflict at hand as well as other moral conflicts caused by scarcity of resources (36, 38) underscore the deeper political nature of the issue. This insight must be taken into account by those who are committed to mitigate moral distress (43, 44) - so that they do not mitigate the political potential in the process.

### 3.2 Limitations

While this paper offers a comprehensive examination of a moral conflict experienced by a professional supporting a family affected by dementia from a cultural, moral, and sociopolitical perspective, it does have certain limitations.

This analysis draws on a case accounted for by the healthcare professionals involved in the conflict. It misses the perspectives of the individual living with dementia, of the informal caregiver, and their daughters. Incorporating their viewpoints would have provided a more comprehensive understanding of the situation and the perceived adequacy of the MT’s action.

The present paper focused on a single scenario encountered during the implementation of an intervention as part of a larger research project. Obviously, no statements on the frequency of the conflict in practice can be derived. This analysis aimed at integrating the ethics of filial duties with the literature on the burden of informal caregiving, with the debate on caring responsibilities, as well as with the works on the political potential of moral distress to improve care provision and the overall healthcare system. It succeeded in doing so, which is why departing from a single observation is not considered to be problematic that the analysis departed from a single observation.

## 4 Conclusion

This paper has delved into a conflict encountered by healthcare professionals supporting family caregivers providing informal dementia care, discussing a situation where professionals, due to the absence of formal care services that could have unburdened an overburdened informal caregiver, reached out to uninvolved adult daughters, requesting them to participate in their parent’s care. The paper highlighted the complex interplay between familial obligations, professional responsibilities, and systemic inadequacies in dementia care provision. The present ethical case analysis assessed whether the healthcare professionals, by contacting the daughters, acted appropriately or not, and to what extent. It was determined that, given the absence of any alternative, the MT’s action was not only appropriate but necessary. The conclusion is that the ethical issue lies not with the actions of the healthcare professionals, but with the state’s failure to provide adequate formal care services. While informal dementia caregiving can be a rewarding experience, the demands on the family caregiver can also be overwhelming, leading in some cases to exploitation. To prevent such exploitation, formal dementia care services (such as nursing homes, memory clinics, day care centers, mobile care services that offer assistance with activities of daily living, respite care services, etc.) must be available as an alternative and to ease the caregiving burden. A state failure to provide such services forces professionals into ethically problematic situations as they attempt to mitigate overwhelming burdens of informal dementia care. In essence, the paper thus may not only contribute to the scholarly literature on ethical conflicts in dementia care but also serve as an argumentative instrument advocating for broader societal and political changes to fairer allocations of care responsibilities, in dementia care and beyond.

## Data Availability

The data that support the findings of this study are available on request from the corresponding author VD. The data are not publicly available due to their containing information that could compromise the privacy of research participants.

## In memoriam

This article is dedicated to the memory of Prof. Alexander Kurz, who died on 29 January 2023.
